# Clinical Characteristics and Immunogenetics of BCGosis/BCGitis in Chinese Children: A 6 Year Follow-Up Study

**DOI:** 10.1371/journal.pone.0094485

**Published:** 2014-04-10

**Authors:** Wenjing Ying, Jinqiao Sun, Danru Liu, Xiaoying Hui, Yeheng Yu, Jingyi Wang, Xiaochuan Wang

**Affiliations:** Department of Clinical Immunology, Children's Hospital of Fudan University, Shanghai, China; Institute of Infectious Diseases and Molecular Medicine, South Africa

## Abstract

In this study, the clinical and immunogenetical features in a cohort of Chinese patients with BCGosis/BCGitis were investigated. For the patients with abnormal immunological functions, Sanger sequencing was used to identify the involved genes. There were 74 confirmed cases of BCGosis/BCGitis during 2007–2012. Classified by infected tissues and organs, no cases only had local infection, 39 patients had a regional infection, 21 patients had a distant infection and 14 patients had a disseminated infection. Thirty-two patients (43.2%) had definitive primary immunodeficiency diseases (PID) and chronic granulomatous disease (CGD) is the most common PID (n = 23, accounted for 71.9% of all PID patients). For CGD patients, based on the anti-tuberculosis treatment, administration of rhIFN-γ resulted in better control of BCGosis/BCGitis. The results indicate that PIDs are associated with susceptibility to BCG disease. For children with BCGosis/BCGitis, immune function evaluation is necessary, and IFN-γ treatment for BCGosis/BCGitis patients with CGD is effective.

## Introduction

The Bacillus Calmette-Guerin (BCG) vaccine has existed for 80 years and is one of the most widely used of all current vaccines. The BCG vaccine has a protective effect against meningitis and disseminated tuberculosis (TB) in children [1]. The World Health Organization (WHO) recommends that all infants in highly endemic countries receive a single dose of the BCG vaccine [2]. For most children, BCG vaccination is harmless. However, infection, even disseminated infection, caused by BCG has occasionally been reported. The incidence of BCG infection is approximately 1∶10,000–1∶1,000,000 [3]. The BCG-induced disease phenotypes were designated as local, regional, distant, or disseminated pattern based on a revised pediatric classification proposed by Hesseling et al. [4]. The former two patterns were conventionally termed as BCGitis and the latter two as BCGosis.

Previous studies suggest that the immunological condition of children is an important factor in BCG infection. In 1995, Casanova et al. [5] reviewed 121 published cases of disseminated BCG infections. They found 61 cases of definitive immunodeficiency disease: 45 cases were severe combined immunodeficiency disease (SCID), 11 cases were chronic granulomatous disease (CGD), 4 cases were acquired immunodeficiency syndrome and 1 case had complete DiGeorge syndrome (CDGS). Norouzi et al. [6] reported that out of 158 patients with BCGosis, 120 of these patients had immunodeficiency disease. These results indicate that immunogenetic factors are critical, as these can lead to BCGosis/BCGitis. However, most of the studies on BCGosis/BCGitis are based on case reports. Until recently, there was no large sample study on the clinical characteristics and immunogenetics of BCGosis/BCGitis.

China remains one of the 22 countries that have a high TB burden that is recognized by the WHO. The prevalence of TB in China fell slightly during the past decade, but the nation still has the world's second-largest population of people with the disease [7]. The Chinese Center for Disease Control and Prevention recommends that all infants receive a single dose of the BCG vaccine immediately after birth. Some infants present with BCGosis/BCGitis after vaccination. So, we conducted this study to clarify the clinical characteristics and to describe the spectrum of primary immunodeficiency diseases (PID) in a cohort of Chinese patients with BCGosis/BCGitis.

## Materials and Methods

### Ethics Statement

This study was approved by the Pediatric Research Ethics Board of Clinical Pharmacology Base, Fudan University. Because all participants are children, we obtained the written informed consent from their parents, who on behalf of the children enrolled in the study.

### Patients

The study began in January 2007 and was completed in December 2012. During this period, after the informed consent forms were obtained, all of the patients who were diagnosed with BCGosis/BCGitis in the Children's Hospital of Fudan University were enrolled in this study. A diagnosis of BCGosis/BCGitis was confirmed by clinical course, dermatological features, pathology, specific polymerase chain reaction (PCR) [8], and/or spoligotyping. The phenotypes of BCGosis/BCGitis were classified as local, regional, distant, and disseminated patterns, as proposed by Hesseling et al. [4].

### Study design

The clinical features of all of the enrolled patients were observed and the basic immunological functions were evaluated. After evaluation of the basic immunological functions, some of the patients were diagnosed with PID. For these patients, the corresponding genes were detected according to their immune phenotype. For the patients with normal basic immunological functions, IL-12/23 and IFN-γ mediated immunity was investigated.

### Routine evaluation of immunological function

The routine evaluation of immunological function involved the analysis of lymphocyte subsets; the detection of immunoglobulins G, A, M, E and complements C3, C4, and CH50; and the analysis of NADPH oxidase activity in neutrophils. Lymphocyte subsets were analyzed using a FACSCalibur flow cytometer (Becton Dickinson, Franklin Lakes, NJ, USA). Anti-CD3, CD4, CD8, CD16, CD56, CD19, and CD45 antibodies (Multitest IMK Kit, Catalog No. 340503, Becton Dickinson, Franklin Lakes, NJ, USA) were used. The immunoglobulins G, A, and M and complement C3 and C4 were detected by nephelometry. The immunoglobulin kit was purchased from Orion Diagnostica Oy (Espoo, Finland). The respiratory burst of neutrophils was determined by measuring hydrogen peroxide production, using DHR analysis [9].

### Whole blood cultures and detection of IFN-γ production

According to a previous study [10], venous blood samples from patients with normal routine immunological functions were collected into heparinized tubes. These blood samples were diluted 1∶2 in RPMI 1640 (Gibco) supplemented with 100 U/ml penicillin and 100 μg/ml streptomycin (Gibco). We dispensed 4.5 ml of the dilute blood sample into 3 wells (1.5 ml/well) of a 24-well plate. The plate was then incubated in an atmosphere containing 5% CO_2_ and 95% air, with the following three different conditions for activation: with medium alone, with LPS (1 ng, Sigma), with LPS (1 ng, Sigma) plus IL-12 (35 ng, R&D). After 48 h, at the end of the incubation stage, the total volume of each well was recovered, centrifuged at 2500 g for 10 min, and the supernatant was stored at −80°C until the analysis. The IFN-γ concentrations were analyzed by ELISA according to the manufacturers' guidelines. The IFN-γ kits were purchased from Invitrogen (Grand Island, NY, USA).

### Direct sequencing

Based on the immune phenotype of these patients, the different genes were sequenced. For patients with CGD, *CYBB*, *CYBA*, *NCF1*, *NCF2*, and *NCF4* genes were sequenced; for patients with SCID, *IL2RG*, *RAG1*, *RAG2*, *JAK3*, *IL7R*, and *LIG4* genes were sequenced; for patients with hyper IgE syndrome (HIES), *STAT3*, *TYK2*, and *DOCK8* genes were sequenced; for patients with hyper IgM syndrome (HIGM), *CD40LG*, *CD40*, *UNG*, and *AICDA* genes were sequenced; for patients with lower IFN-γ production, *IL12RB1* and *IFNGR1* genes were sequenced.

Genomic DNA was isolated from PBMCs using the RelaxGene Blood DNA System (Tiangen Biotech, Beijing, China) according to the manufacturer's instructions and amplified by PCR using synthetic oligonucleotide primers. The primer sequences were based on human genomic sequences and are available upon request. PCR products were purified by Performa DTR Gel Filtration Cartridges and directly sequenced by ABI Prism BigDye terminators. All of the entire coding regions were covered. Both strands were sequenced.

## Results

### Overview of the cases

There were 74 confirmed cases of BCGosis/BCGitis during the study period: 59 (79.7%) patients were boys and 15 (20.3%) patients were girls. These patients were all healthy at birth and had no contact history of TB. All patients were vaccinated with BCG within two days after birth. Among these 74 children, 32 (43.2%) had definitive PID, including 23 (31.1%) cases with CGD, 2 (2.7%) case with SCID, 2 (2.7%) cases with HIGM, 1 (1.4%) case with HIES, and 4 (5.4%) cases with Mendelian susceptibility to mycobacterial diseases (MSMD).

### Clinical characteristics

#### Age of onset

Among the 74 patients, the median age of onset of BCGosis/BCGitis was 3.6 months old (range: 20 days to 4 years). Among the 32 patients with definitive PID, 22 (68.8%) patients presented with this condition within 1 year of vaccination, and the median age of onset is 3 months old (range: 20 days to 4 years). Among the 42 patients without definitive PID, the median age of onset is 4 months old (range: 1 month to 2 years). There is no significant difference in the age of onset of BCGosis/BCGitis between these two groups.

#### BCG disease classification

**Figure 1:**
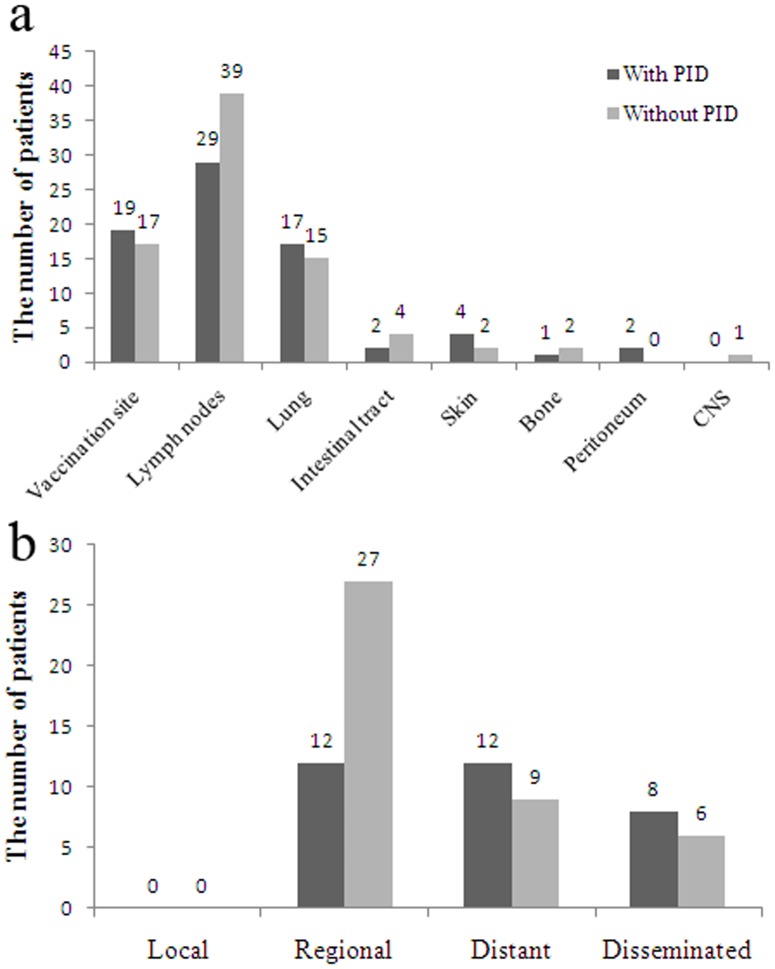
Clinical characteristics of 74 patients with BCGosis/BCGitis. a. infected tissues and organs; b. classification according to the infected tissues and organs. PID: primary immunodeficiency disease. CNS: central nervous system.

In the previous study [4], BCG disease was classified as local, regional, distant, and disseminated. Local or regional BCG disease was diagnosed upon confirmation of *M. bovis* BCG from fine-needle aspiration or swab samples of pus; distant or disseminated disease was diagnosed upon isolation of *M. bovis* BCG from respiratory isolates in children with respiratory symptoms or from other distant sites. Among the 74 patients, the most common tissues and organs that were affected are lymph nodes, vaccination site, and lung, regardless of whether patients had PID or not (Figure 1a). According to the above mentioned BCG disease classification, no case only had local infection, 39 (52.7%) patients had regional infection, 21 (28.4%) patients had distant infection and 14 (18.9%) patients had disseminated infection (Figure 1b). Classified by with or without definitive PID, 62.5% (20/32) patients with PID had distant or disseminated infection, and only 35.7% (15/42) patients without PID had distant or disseminated infection (Figure 1b). The results indicated that patients with PID usually had more severe BCG infection than patients without PID.

### Immunological characteristics

The immunological functions of all 74 patients were evaluated. Twenty-eight patients had abnormal immunological functions. The immunological characteristics of all the 74 patients are shown in Table 1. Based on the immunological phenotype and clinical characteristic data, they were diagnosed with CGD (n = 23), SCID (n = 2), HIGM (n = 2), and HIES (n = 1), respectively. For the two SCID patients, we also detected their human immunodeficiency virus (HIV) status, because they had lower T cells counts than normal. Both are negative.

**Table 1:** Routine immunologic function evaluation in 74 BCGosis/BCGitis patients.

	without PID* (n = 42)	with CGD* (n = 23)	with HIGM* (n = 2)	with HIES* (n = 1)	with SCID* (n = 2)	with MSMD* (n = 4)
Lymphocyte subsets						
CD3+(%)	51–77	55–71	82; 72	94	9; 11	48–57
CD3+CD4+(%)	35–55	23–44	21; 22	52	4; 3	29–27
CD3+CD8+(%)	13–36	31–45	49; 45	27	4; 4	17–27
CD16+CD56+(%)	5–35	8–17	15; 17	2	2.5; 3.2	6–7
CD19+(%)	3–17	7–21	3; 5	4	65; 71	40–31
Immunoglobulin level						
IgG(g/L)	3.7–24.8	8.6–26	1.2; 1.75	24.6	0.98; 0.57	7.2–11.3
IgA(g/L)	0.1–2.24	0.81–3.02	1.28; 2.49	0.38	0.067; 0.03	0.23–0.98
IgM(g/L)	0.09–3.24	1.02–3.27	6.76; 55.34	3.08	0.04; 0.02	0.24–1.57
IgE(kU/L)	2.6–390	24.5–990	<2; 3.96	129000	<2	7.14–206
DHR analysis# (SI*)	>100	<10	>100	>100	>100	>100

*PID: primary immunodeficiency disease; CGD: chronic granulomatous disease; HIGM: hyper IgM syndrome; HIES: hyper IgE syndrome; MSMD: Mendelian susceptibility to mycobacterial diseases. SI: stimulation index.

# DHR analysis: The comparison was based on a stimulation index, which was defined as mean channel fluorescence intensity of PMA-stimulated neutrophils over mean channel fluorescence intensity of unstimulated neutrophils.

### Detection of IFN-γ production

The production of IFN-γ in 46 patients with normal immune function was evaluated. We compared the production of IFN-γ in diluted whole blood after stimulation with medium alone, with LPS, and with LPS plus IL-12. We supplemented the medium with IL-12, as it is a potent inducer of IFN-γ. Four patients showed significantly lower IFN-γ concentrations (37.30–37.77 pg/ml) in the supernatant after stimulation with medium alone, and IFN-γ concentrations did not significantly increase after stimulation with LPS (39.30–41.85 pg/ml) or with LPS plus IL-12 (48.10–61.59 pg/ml). Compared with these 4 patients, the remaining 42 patients showed higher IFN-γ concentrations in the supernatant after stimulation with medium alone (mean, 93.75 pg/ml) and with LPS (mean, 98.65 pg/ml), and IFN-γ concentrations had significantly increased after stimulation with LPS plus IL-12 (mean, 921.51 pg/ml).

### Gene sequencing

Gene sequencing was performed for 28 patients with definitive PID and for 4 patients with lower IFN-γ production. Mutations were found in 26 patients. For 23 CGD patients, mutations in the *CYBB*, *CYBA*, *NCF1*, *NCF2* and *NCF4* genes were detected. Among the 23 CGD patients, 17 patients had a *CYBB* mutation, 1 patient had a *CYBA* mutation, 2 patients had a *NCF2* mutation, and no mutation was found in 3 cases. For 2 SCID patients, mutations in *IL2RG* were found. For 2 HIGM patients, *CD40LG*, *CD40*, *UNG*, and *AICDA* genes were sequenced. For 1 HIES patient, *STAT3*, *TYK2*, and *DOCK8* genes were sequenced. However, no mutation was found. For the 4 patients with lower IFN-γ production, we sequenced *IL12RB1* and *IFNGR1* genes. Two patients had a mutation in *IFNGR1* gene and the other 2 patients had a mutation in *IL12RB1* gene. The details of all the mutations are shown in Table 2.

**Table 2:** Details of gene mutations in 26 BCGosis/BCGitis patients with primary immunodeficiency.

Patient NO.	Gene	Mutation type	CDS level change	Protein level change
1	*CYBB*	deletion	c.1177delA	p.G393fsX404
2	*CYBB*	deletion	c.343–344delCA	p.H115fsX121
5	*CYBB*	deletion	c.76–77delTT	p.F26fsX33
6	*CYBB*	missense	c.1082G>T	p.W361L
8	*CYBB*	missense	c.1366G>A	p.D456N
9	*CYBB*	missense	c.665A>G	p.H222R
15	*CYBB*	nonsense	c.676C>T	p.R226X
23	*CYBB*	nonsense	c.1320C>A	p.Y440X
26	*CYBB*	nonsense	c.370G>T	p.E124X
28	*CYBB*	nonsense	c.676C>T	p.R226X
32	*CYBB*	nonsense	c.388C>T	p.R130X
42	*CYBB*	splice 3′	c.253-3A>G	del. Exon 4
51	*CYBB*	splice 5′	c.252+5G>A	del. Exon 3
57	*CYBB*	splice 5′	c.1150–1151+2delAAGT	del. Exon 9
59	*CYBB*	splice 5′	c.252+5G>A	del. Exon 3
63	*CYBB*	splice 5′	c.1152G>C	del. Exon 9 K384N
65	*CYBB*	splice 5′	c.252+2dupT	del. Exon 3
71	*CYBA*	nonsense	c.7C>T	p.Q3X
18	*NCF2*	deletion	c.1130–1135delACATGG	p.Asp377-Met378del
47	*NCF2*	missense	c.137T>G	p.M46R
14	*IL2RG*	missense	c.314A>G	p.Y105C
60	*IL2RG*	deletion	c.432–433delGA	p.Q144fsX22
35	*IL12RB1*	missense	c.1094T>C	p.M365T
48	*IL12RB1*	missense	c.1094T>C	p.M365T
37	*IFNGR1*	missense	c.655G>A	p.G219R
54	*IFNGR1*	missense	c.1400T>C	p.L467P

### Treatment and outcome

Forty-two patients without definitive PID received routine anti-TB treatment (Isoniazid, rifampicin and ethambutol). Among the 42 patients, 2 died from disseminated TB during therapy, and the remaining 40 patients were cured after 1 year of treatment.

Among the 23 CGD patients, 19 received routine anti-TB treatment, 1 was lost to follow-up, and 3 refused anti-TB treatment and died. Among the 19 patients received routine anti-TB treatment, 7 received recombinant human interferon-γ (rhIFN-γ) treatment (1 MIU/m^2^, twice a week) together with anti-TB treatment. In all the 7 patients, BCGosis/BCGitis was cured after 1 year of treatment. Because the remaining 12 patients were not diagnosed with CGD when BCGosis/BCGitis was diagnosed, they only received a routine anti-TB treatment. After treatment for more than 1 year, all the 12 patients were not cured and 3 died. When the remaining 9 patients were transferred to our center, they were diagnosed with CGD. They received treatment with routine anti-TB drugs and rhIFN-γ. After 1 year of treatment, BCGosis/BCGitis was cured in all the 9 patients.

Among the 4 patients with lower IFN-γ profuction, 2 cured after 2 years treatment with routine anti-TB drugs and rhIFN-γ, 1 died from disseminated TB during therapy, and 1 is still treated. Two SCID patients and 1 HIES patient also died from disseminated TB during therapy. In addition, 2 HIGM patients had received anti-TB treatment for more than 2 years, and BCGosis/BCGitis was not cured.

## Discussion

The BCG vaccine has existed for 80 years and is one of the most widely used of all current vaccines, reaching >80% of neonates and infants in countries where it is part of the national childhood immunization program [1]. The BCG vaccine is widely used, and the safety of this vaccine has not been a serious issue until recently. Complications that arise from BCG vaccination are uncommon. Less than one in 1000 vaccinated individuals develop severe local reactions, and serious disseminated disease develops in less than one in a million cases [3]. There is a concern that use of the vaccine in people who are immunocompromised may result in an infection that is caused by BCG itself. The data reported by Casanova et al. [5] and Norouzi et al. [6] showed that 50% to 76% of BCG-infected patients had immunodeficiency. China is a high-TB-burden country. All full-term neonates are recommended to receive a single dose of BCG vaccine immediately after birth. With the improvement of medical technology, more children are being diagnosed with BCGosis/BCGitis in China. However, the data on the clinical characteristics and immunological conditions of these patients are lacking.

To investigate whether immunodeficiency is the main cause of BCGosis/BCGitis, we conducted the present study. The results showed that more than 40% patients with BCGosis/BCGitis had definitive PID. The proportion of patients with immunodeficiency is lower than in previous studies [5], [6]. Moreover, the proportion of each type of PID is different. Casanova et al. [5] and Norouzi et al. [6] showed that SCID is the most common form of immunodeficiency in children with BCGosis/BCGitis. However, our study suggested that CGD is the most common form of immunodeficiency. This difference may be explained by the following reasons: 1. Most patients had disseminated BCG infection in the above mentioned two studies. However, only 14 patients had disseminated BCG infection in our study. Most of our patients had regional and distant infections. The difference in the types of PID between the previous study and our study may be explained by a difference in the severity of BCG infection; 2. Only a few hospitals have the ability to diagnose SCID in China. Children with SCID usually have more serious infections, and many of them die in local hospitals from complications of serious infection. These patients cannot obtain a clear diagnosis. In addition, we estimated that the incidence of SCID is lower in Chinese people. Accurate epidemiological data need further investigation.

Among the 74 patients with BCGosis/BCGitis, 23 had CGD. These results indicate that CGD patient are susceptible to BCG infection. CGD is a form of immunodeficiency that affects phagocytic leukocytes [11]. In CGD patients, leukocyte NADPH oxidase is inactive as a result of mutations in any of five genes that encode essential subunits of the enzyme, which comprise the structural components of NADPH oxidase, including gp91phox, p22phox, p47phox, p67phox and p40phox [12]–[14]. These molecular defects result in susceptibility to *Mycobacterium*. In our study, we found that BCGosis/BCGitis in 87% of CGD patients occurred within 1 year after BCG vaccination; 1 patient was 4 years old and the median age of onset was 3 months of age. The results suggest that the age of onset of BCGosis/BCGitis in most CGD children is earlier; however, it should be noted that in a few CGD children, the disease onset occurred later. Among the 23 CGD patients, gene mutations were found in 20 patients, including 17 CYBB mutations, 1 CYBA mutation and 2 NCF2 mutations. A correlation between gene mutations and the severity of BCG infection was not found.

Recent work showed that the IL-12/IFN-γ signaling pathway plays an important role in immunity against mycobacterial infection. The disease caused by molecular defects in the IL-12/IFN-γ signaling pathway is called MSMD. Currently, many cases of IL-12/IFN-γ signaling pathway defects have been found in a number of countries [15], [16], including 2 cases with an *IL12RB1* gene mutation in China [15]. In this study, 46 patients were not found to have PID by routine immunological function evaluation. These patients had detectable IFN-γ production. Among the 46 patients, 4 had lower IFN-γ production. Two had *IL12RB1* mutation and 2 had *IFNGR1* mutation. Both *IL12RB1* mutations are homozygous. For *IFNGR1* mutation, one is homozygous mutation and the other is heterozygous mutation. The homozygous mutation site has been reported in the Human Gene Mutation Database (http://www.hgmd.org/). In the NCBI database (http://www.ncbi.nlm.nih.gov/projects/SNP/), the heterozygous mutation site has been reported as an SNP (rs1887415). However, our results showed that the patient with an *IFNGR1* heterozygous mutation had lower IFN-γ production and disseminated BCG infection. These results suggest that the site may be associated with susceptibility to mycobacterial infection. Further research into whether the site has a mutation or an SNP is needed.

A randomized, double-blind, placebo-controlled study showed that IFN-γ therapy is an effective and well-tolerated treatment for CGD patients [17]. Ahlin A., et al. found that IFN-γ treatment of patients with CGD is associated with augmented production of nitric oxide by polymorphonuclear neutrophils [18]. In our study, patients with CGD and MSMD were treated with rhIFN-γ and anti-TB drugs. The results showed that with anti-TB treatment, administration of rhIFN-γ provided better control of BCGosis/BCGitis. However, the results need to be verified by a large-sample, randomized, double-blind, placebo-controlled study.

In summary, BCGosis/BCGitis is an important indicator of immunodeficiency. CGD is the most common PID in children with BCGosis/BCGitis in China. For children with BCGosis/BCGitis, immune function evaluation is necessary, and IFN-γ treatment for BCGosis/BCGitis patients with CGD is effective.
